# Supplementary material for: Epidemiological investigation and physician awareness regarding the diagnosis and management of Q fever in South Korea, 2011 to 2017
Source: PLoS Negl Trop Dis. 2021 Jun 2;15(6):e0009467. doi: 10.1371/journal.pntd.0009467 (PMC8202952; doi:10.1371/journal.pntd.0009467)
Supplement: S1 Appendix — (DOCX) [file pntd.0009467.s001.docx]

**S1 Appendix. Survey of physician awareness for Q fever diagnosis and management.**

**Ⅰ. Physician characteristics**

1. How many years of clinical experience do you have since you became an internist?

- less than 5 years
- 5 to 9 years
- 10 to 14 years
- more than 15 years

2. What type of hospital do you work at?

- university hospital
- non-university hospital

3. Where is the area of your affiliated hospital?

- Seoul
- Busan
- Dae-gu
- Incheon
- Gwangju
- Daejeon
- Ulsan
- Gyeonggi
- Gangwon
- Chungbuk_sejong
- Chungnam
- Jeonbuk
- Jeonnam
- Gyeongbuk
- Gyeonnam
- Jeju

4. Have you ever requested a Q fever antibody or PCR test? How many cases have you tested for Q fever?

- No/None at all
- Less than 10 cases
- 10 to 30 cases
- more than 30 cases

5. For which patients did you request a Q fever test?

(Please select all applicable items)

- Never requested a Q fever test
- A febrile patient with occupational risks, such as veterinarians, livestock raisers, butchers, meat processors
- A febrile patient with a history contact with animals such as cattle, sheep, goats
- A febrile patient with a history of visiting a barn or farm
- A patient who had a fever of unknown origin for more than three weeks without an epidemiologic risk factor
- A patient who has an unexplained fever within two weeks
- A patient with a fever and modest increase in transaminase (AST/ALT 200 <IU/L) levels
- A patient with atypical pneumonia
- A patient with culture-negative endocarditi

6. How many patients have you diagnosed with Q fever?

- None at all
- 1 to 2 patients have been diagnosed with Q fever
- 3 to 5 patients have been diagnosed with Q fever
- 6 to 10 patients have been diagnosed with Q fever
- More than 10 patients have been diagnosed with Q fever

**Ⅱ. Diagnosis and management of acute Q fever**

7. If the patient that was suspected to have acute Q fever had a negative result in the first serologic test, did you perform a follow-up serologic test at the convalescent stage of illness?

- Follow-up serologic tests were rarely performed
- If possible, a 2^nd^ serologic test for Q fever was performed
- A patient was strongly recommended to undergo a 2^nd^ serologic test for Q fever

8. If the serologic test is initially negative result in suspected patients with acute Q fever, when do you perform the second serologic test for Q fever?

- The 2nd serologic test for Q fever is rarely performed
- 1-2 weeks after the first serologic test
- 2-3 weeks after the first serologic test
- 3-4 weeks after the first serologic test
- 4-6 weeks after onset of symptom

9. Choose the reasons why the second serologic test for Q fever was not performed in suspected patients who had initially negative serologic result at the acute stage of the illness.

(Please select all applicable items)

- They were diagnosed with other diseases
- Unable to follow-up with the patient following clinical improvement
- The need for a follow-up test to identify Q fever was lower following symptom improvement

**Ⅲ. Diagnosis and management of chronic Q fever**

10. How do you screen for the risk factors of persistent localized Q fever after acute Q fever diagnosis?

- Have no experience in treating patients with acute Q fever
- Clinical and serologic follow-ups are not considered after the patients have improved
- Perform echocardiography to identify valve abnormalities as much as possible
- Perform chest or abdominal CT scan to identify vascular abnormalities as much as possible
- Whether a patient is at risk for chronic Q fever is based on clinical assessments that do not involve echocardiographs or CT scans

11. Do you repeat clinical assessments and serologic tests for more than 6 months to identify persistent localized Q fever after acute Q fever was diagnosed?

- No, clinical assessment and serologic tests are not repeated after acute Q fever is confirmed and recovered
- Yes, clinical assessments and serologic tests are repeated once or twice after acute Q fever is confirmed and recovered
- Yes, clinical assessments and serologic tests are repeated for at least 6 months after acute Q fever is confirmed and recovered

12. Have you ever treated a patient with persistent localized Q fever such as endocarditis or vascular infection?

- No
- Yes

13. Have you ever performed a serologic or PCR test for Q fever in patients with culture-negative endocarditis, infected aneurysms, or vascular graft infections?

- No
- Yes
