# Supplementary figures and images for: Epidemiological investigation and physician awareness regarding the diagnosis and management of Q fever in South Korea, 2011 to 2017
Source: PLoS Negl Trop Dis. 2021 Jun 2;15(6):e0009467. doi: 10.1371/journal.pntd.0009467 (PMC8202952; doi:10.1371/journal.pntd.0009467)

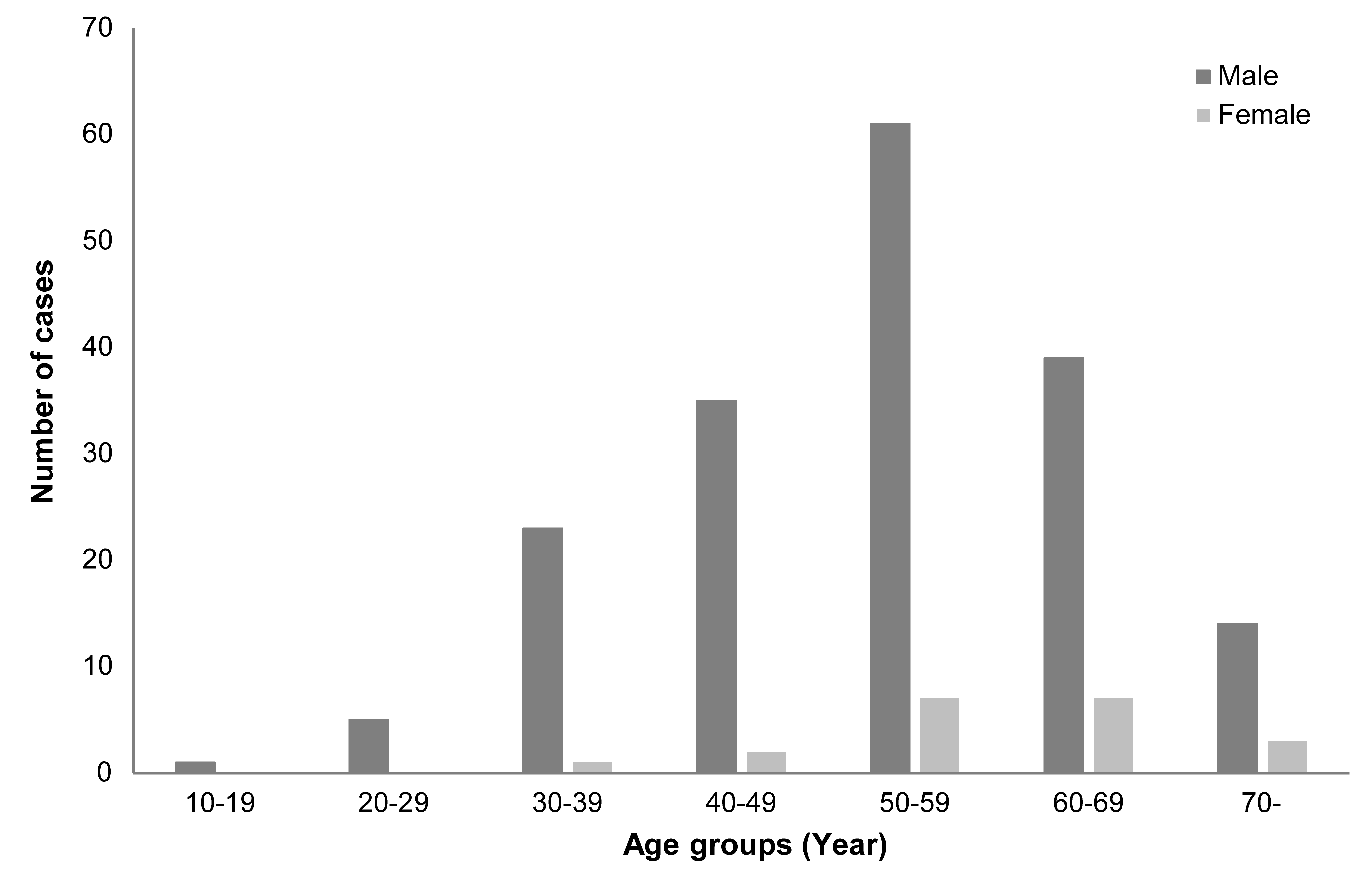

Supplement: S1 Fig — (TIF) [file pntd.0009467.s002.tif]
