# Supplementary material for: Epidemiological investigation and physician awareness regarding the diagnosis and management of Q fever in South Korea, 2011 to 2017
Source: PLoS Negl Trop Dis. 2021 Jun 2;15(6):e0009467. doi: 10.1371/journal.pntd.0009467 (PMC8202952; doi:10.1371/journal.pntd.0009467)
Supplement: S1 Table — (DOCX) [file pntd.0009467.s003.docx]

**S1 Table. Temporal distribution of Q fever cases from 2011 to 2017.**

|  | Jan | Feb | Mar | Apr | May | Jun | Jul | Aug | Sept | Oct | Nov | Dec |
| --- | --- | --- | --- | --- | --- | --- | --- | --- | --- | --- | --- | --- |
| 2011 | 0 | 0 | 0 | 1 | 0 | 2 | 3 | 1 | 0 | 0 | 0 | 1 |
| 2012 | 0 | 1 | 1 | 1 | 2 | 2 | 2 | 0 | 0 | 0 | 1 | 0 |
| 2013 | 0 | 0 | 1 | 0 | 0 | 2 | 2 | 2 | 1 | 1 | 1 | 1 |
| 2014 | 3 | 1 | 0 | 0 | 1 | 1 | 0 | 0 | 0 | 0 | 0 | 2 |
| 2015 | 2 | 1 | 7 | 1 | 1 | 0 | 6 | 0 | 3 | 2 | 2 | 2 |
| 2016 | 2 | 3 | 5 | 3 | 3 | 10 | 6 | 3 | 11 | 7 | 20 | 8 |
| 2017 | 1 | 5 | 8 | 6 | 10 | 13 | 11 | 11 | 13 | 4 | 7 | 7 |
