# Supplementary material for: Epidemiological investigation and physician awareness regarding the diagnosis and management of Q fever in South Korea, 2011 to 2017
Source: PLoS Negl Trop Dis. 2021 Jun 2;15(6):e0009467. doi: 10.1371/journal.pntd.0009467 (PMC8202952; doi:10.1371/journal.pntd.0009467)
Supplement: S2 Table — (DOCX) [file pntd.0009467.s004.docx]

**S2 Table. The results of questionnaire other than key questions.**

| Questions and answers | Response rate (%) |
| --- | --- |
| Q1) How many years of clinical experience do you have since you became an internist? | |
| Less than 5 years | 18.5 |
| 5 to 9 years | 32.0 |
| 10 to 14 year | 23.3 |
| more than 15 years | 26.2 |
| Q2) What type of hospital do you work at? (select all applicable items) | |
| University hospital | 80.6 |
| Non-university hospital | 19.4 |
| Q3) Where is the area of your affiliated hospital? | |
| Seoul | 35.0 |
| Busan | 3.9 |
| Daegu | 3.9 |
| Incheon | 6.8 |
| Kwangju | 5.8 |
| Daejeon | 1.9 |
| Ulsan | 1.0 |
| Gyeonggi | 26.2 |
| Gangwon | 1.9 |
| Chungbuk | 1.9 |
| Chungnam | 1.0 |
| Jeonbuk | 1.9 |
| Jeonnam | 1.0 |
| Gyeongnam | 6.9 |
| Jeju | 1.0 |
| Q4) If the serologic test is initially negative result in suspected patients with acute Q fever, when do you performed the 2nd serologic test for Q fever? | |
| The 2nd serologic test for Q fever is rarely performed | 35.9 |
| 1-2 weeks after the first serologic test | 13.6 |
| 2-3 weeks after the first serologic test | 26.8 |
| 3-4 weeks after the first serologic test | 9.6 |
| 4-6 weeks after symptom onset | 14.4 |
| Q5) Have you ever treated a patient with chronic Q fever such as endocarditis or vascular infection? | |
| No | 85.4 |
| Yes | 14.6 |
