# Supplementary material for: Epidemiological investigation and physician awareness regarding the diagnosis and management of Q fever in South Korea, 2011 to 2017
Source: PLoS Negl Trop Dis. 2021 Jun 2;15(6):e0009467. doi: 10.1371/journal.pntd.0009467 (PMC8202952; doi:10.1371/journal.pntd.0009467)
Supplement: Attachment — Submitted filename: Response to reviewers.docx [file pntd.0009467.s005.docx]

Manuscript ID: PNTD-D-20-01239

**Title:** Epidemiological investigation and physician awareness regarding the diagnosis and management of Q fever in South Korea, 2011 to 2017

Thank you for your constructive comments and criticism on our manuscript. The manuscript has been carefully rechecked and appropriate changes have been made in accordance with the reviewers’ suggestions. We believe that the quality of the manuscript is has significantly improved after incorporating the reviewers’ suggestions. Our point-by-point responses to the reviewers are provided below. We hope that the revised manuscript is now suitable for publication in your journal. We look forward to your response.

Sincerely,

Jung Yeon Heo, MD

Department of Infectious Diseases, Ajou University School of Medicine, 164, Worldcup-ro, Youngtong-gu, Suwon 16499, Republic of Korea.

Tel: 82-31-219-7818; E-mail address: jyeon78@naver.com

**Detailed Response to Reviewers**

**Notes:** The reviewers’ comments are in boxes, and our responses are in plain text.

**Methods**

**Reviewer #1:**

No response

**Reviewer #2:**

**Comment #1**

Although the definition of confirmed and probable case of acute Q fever and chronic Q fever seem clear in this section, it is not completely congruent with the results section and it should be reviewed (see comments below).

**Authors’ response to comment #1>**

Thank you for your comment. We have defined Q fever in the Methods section and accordingly modified the Results section as follows:

“Based on the laboratory criteria, acute Q fever was defined as confirmed or probable in patients with symptoms consistent with acute febrile illness such as flu-like symptoms, hepatitis, or pneumonia. Laboratory-confirmed cases of acute Q fever were defined as follows: 1) a ≥4-fold increase in immunoglobulin G (IgG) titer to phase II antigen between paired serum samples or 2) positive polymerase chain reaction (PCR) results for *C*. *burnetii* nucleic acid in the clinical specimens. If the IgG titer to phase II antigen in the acute phase was ≥1:256 and the follow-up serum sample from the convalescent phase was not collected, the case was considered a laboratory probable case.”

**Comment #2**

The paragraph explaining the survey is redundant since all data are available in Table 4 and supplementary material, this paragraph could be summarized.

**Authors’ response to comment #2>**

Thank you for your detailed comment. We have summarized the paragraph explaining the survey, as follows:

“We administered an online survey to physicians, including infectious disease specialists, who were registered with the Korean Society of Infectious Disease from December 17 to December 31, 2018. The survey consisted of questions to investigate physicians’ awareness regarding how to accurately diagnosis and manage Q fever. The questionnaire contained 12 questions regarding physicians’ clinical careers, place of work, experience in treating patients with Q fever, and knowledge regarding its diagnosis and management. The detailed questions and their answers are presented in S1 Appendix.”

**Comment #3**

The authors say that the Institutional Review Board waived the requirement for written consent. Tis fact is hugely surprising, since Ethics Committees require it when it is necessary to consult the medical personal history of patients. To clarify this fact the authors could explain how the information was extracted avoiding the consultation of these documents.

**Authors’ response to comment #3>**

Thank you for your comment. The study was approved by the Institutional Review Board because it was stipulated that the patients’ personal information will be kept confidential during the process of collecting and analyzing clinical data and writing the manuscript. In addition, the Institutional Review Board waived the requirement for obtaining written consent from the patients because this study was conducted retrospectively using anonymized data that were all previously collected.

**Reviewer #3**

**Comment #1**

The methods are well described and clear to the reader.

A few minor comments in this section - I am not aware of the definition of "probable case" as defined by the authors by a single IgG phase 2 >256. This titer might as well represent a past infection, and this should be stated to my opinion.

**Authors’ response to comment #1>**

Given that a single serological test has diagnostic limitations in confirming infectious diseases, the reviewer’s opinion makes sense. However, many patients with suspected acute Q fever underwent only a single serological test. Titers of phase II IgG ≥1:200 have been considered significant for the diagnosis of acute Q fever in previous studies [1, 2]. The definition of “probable case” in our study was determined based on a French recommendation by Raoult et al. and case definition and classification for acute and chronic Q fever propsosed by the US CDC [3, 4].

1. Fournier PE, Marrie TJ, Raoult D. 1998. Diagnosis of Q fever. J Clin Microbiol 36:1823–1834.

2. Dupont HT, Thirion X, Raoult D. 1994. Q fever serology: cutoff determination for microimmunofluorescence. Clin Diagn Lab Immunol 1:189 –196.

3. Anderson A, Bijlmer H, Fournier PE, Graves S, Hartzell J, Kersh GJ, et al. Diagnosis and management of Q fever--United States, 2013: recommendations from CDC and the Q Fever Working Group. MMWR Recomm Rep. 2013; 62: 1-30.

4. Eldin C, Melenotte C, Mediannikov O, Ghigo E, Million M, Edouard S, et al. From Q Fever to Coxiella burnetii Infection: a Paradigm Change. Clin Microbiol Rev. 2017; 30: 115-190.

**Comment #2**

In addition, the survey to evaluate physicians' awareness of Q fever is in the appendix, and I think it is not necessary to write so many examples of questions in so many details.

**Authors’ response to comment #2>**

Thank you for your thoughtful comment. We have summarized the paragraph explaining the survey, as follows:

“We administered an online survey to physicians, including infectious disease specialists, who were registered with the Korean Society of Infectious Disease from December 17 to December 31, 2018. The survey consisted of questions to investigate physicians’ awareness regarding how to accurately diagnosis and manage Q fever. The questionnaire contained 12 questions regarding physicians’ clinical careers, place of work, experience in treating patients with Q fever, and knowledge regarding its diagnosis and management. The detailed questions and their answers are presented in S1 Appendix.”

**Results**

**Reviewer #1**

**Comment #1**

Concerning acute Q fever, the table reported is the one known in the literature Concerning "chronic Q fever". It must be clarified, as the authors are beginning to do. This term is becoming obsolete and that it must be replaced by resistant focalised infections, which means that the risk factors of its forms do not constitute risk factors but fixing factors. Valvulopathies or aneurysms are not risk factors for chronic Q fever but risk factors for chronic infection of aneurysms or valvulopathies.

**Authors’ response to comment #1>**

We have deliberated on your criticism for the term “chronic Q fever.” We also agree that chronic Q fever is not an appropriate term, and some experts suggest using persistent localized infection instead of the term chronic Q fever. Similarly, acute *C. burnetii* infection or primary *C. burnetii* infection would be a better understanding of the clinical forms of human Q fever. However, most clinicians have become accustomed to the term Q fever rather than the term *C. burnetii* infection. Furthermore, the terms acute and chronic Q fever are generally used in most infectious diseases textbooks such as Harrison’s internal medicine. Therefore, we had used the term “persistent localized infection” together with chronic Q fever in the introduction section. After careful consideration, we have decided not to change the terms acute or chronic Q fever to acute or persistent localized infection of *C. burnetii*.

Although the features of acute Q fever in this study might be well known in endemic areas, these features might not be known to other physicians in some countries that are becoming endemic areas, such as South Korea. We would like to suggest that these clinicoepidemiological features and low awareness among clinicians should be considered to promote the appropriate diagnosis and management of Q fever in our study.

**Comment #2**

It would be interesting to reflect on the importance of Q fever in endocarditis with negative blood cultures in Korea and to see if there are systematic studies carried out for this purpose and this could be a suggestion in the discussion to make a systematic Coxiella burnetii serology or PCR on valves of endocarditis, negative blood culture which allows a true evaluation of the incidence. In the same way, the revelation by PET scanner of a fixation on a vascular prosthesis or an aneurysm is a formal indication to carry out an examination to detect Q fever. These elements could be usefully added.

**Authors; response to comment #2>**

Thank you for your meaningful comment. As suggested, we have included the following paragraph in our manuscript discussion.

“In South Korea, vascular infection was a more common clinical manifestation than endocarditis in patients with chronic Q fever. It was known that endocarditis is most common manifestation among patients with chronic Q fever. In some studies, *C. burnetii* infection was the most frequently identified etiology in patients with culture-negative endocarditis [24,25]. In this study, Q fever endocarditis was only confirmed in 6 cases. However, culture-negative endocarditis cases accounted for about a quarter of endocarditis cases in South Korea [26,27]. This suggests that Q fever endocarditis may be an underestimated disease in South Korea, and serological tests or PCR tests for *C. burnetii* should be more actively performed in cases of culture-negative endocarditis.”

**Comment #3**

The whole discussion about the knowledge of Korean physicians is not of major interest to investigators in other countries. Concerning the different scenarios, Table 1 is not really of interest and could be deleted over, as could Table 4.

**Authors' response to comment #3>**

Thank you for your comment. As we mentioned above, some physicians in other countries who understood Q fever were not interested in the awareness and knowledge of Korean physicians. However, the disease courses of human Q fever are quite different from those of other bacterial infectious diseases. *C. burnetii* infection can persist after primary infection; hence, appropriate follow-up or prevention strategies are required for at-risk patients. This is not known to many physicians in other countries as well as in South Korea. Thus, we think that this study provides useful data for some countries or physicians to promote the appropriate management in patients with suspected or confirmed *C. burnetii* infection.

**Reviewer #2**

**Comment #1**

It is important to confirm that the 200 patients are different, and no chronic Q fever case was included also as an acute Q fever case, since the study period could lead to this mistake.

**Authors' response to comment #1>**

Thank you for your detailed comment. When the clinical information of the 200 patients was collected, we confirmed that all the patients with Q fever were included only once in the analysis and we avoided duplicate entry in the analysis using data on patients’ age, sex, and postal code. We have also added the following sentence in the *Clinical characteristics of patients with acute Q fever* subsection in the Results section:

“None of the patients with acute Q fever progressed to persistent localized infection or died.

”

**Comment #2**

Acute Q fever cases: the diagnosis criteria are very confusing regarding acute cases and in the present text form it seems that some patients had only a clinical diagnosis. The authors should re-write this paragraph and clarify which cases were diagnosed with a single serological test, seriated serological tests or only clinical criteria. These data should also be congruent with those shown in Table 2.

**Authors’ response to comment #2>**

We apologize for the confusion. All cases were classified according to the clinical and laboratory criteria. Thus, we have clearly presented diagnostic criteria in the method section as follows:

“Based on the laboratory criteria, acute Q fever was defined as confirmed or probable in patients with symptoms consistent with acute febrile illness such as flu-like symptoms, hepatitis, or pneumonia. Laboratory-confirmed cases of acute Q fever were defined as follows: 1) a ≥4-fold increase in immunoglobulin G (IgG) titer to phase II antigen between paired serum samples or 2) positive polymerase chain reaction (PCR) results for *C*. *burnetii* nucleic acid in the clinical specimens. If the IgG titer to phase II antigen in the acute phase was ≥1:256 and the follow-up serum sample from the convalescent phase was not collected, the case was considered a laboratory probable case.”

In addition, we have revised in the *Clinical characteristics of acute Q fever* subsection in the Results section and Table 2 as follows:

“The clinical manifestations were primarily hepatitis (118 cases, 64.5%), isolated febrile illness (44 cases, 24.0%), and pneumonia with or without hepatitis (17 cases, 9.3%). Among the patients with acute Q fever, 116 (63.4%) patients underwent serological tests using paired sera collected at least twice during the acute and convalescent phase. Therefore, the remaining 67 (36.6%) cases in which only a single serological test was performed were classified as probable acute Q fever cases. Fifty (27.3%) patients with acute Q fever had seroconversion, from seronegative in the acute stage to seropositive in the convalescent stage.”

| ^Variables^ | | ^Acute Q fever (n=183)^ |
| --- | --- | --- |
|  |  | ^No. (%)^ |
| ^Clinical symptoms^ | |  |
|  | ^Fever^ | ^175 (95.6)^ |
|  | ^Chill^ | ^101 (55.2)^ |
|  | ^Myalgia^ | ^107 (58.5)^ |
|  | ^Headache^ | ^88 (48.1)^ |
|  | ^Fatigue/weakness^ | ^55 (30.1)^ |
|  | ^Cough^ | ^36 (19.7)^ |
| ^Laboratory findings^ | |  |
|  | ^WBC (/mm3), median (IQR)^ | ^7,920 (5,287–11,202)^ |
|  | ^Platelet (×103/mm3), median (IQR)^ | ^199 (127–371)^ |
|  | ^CRP (mg/dL), median (IQR)^ | ^7.9 (2.6–12.3)^ |
|  | ^ALT (IU/L), median (IQR)^ | ^74 (37–128)^ |
|  | ^LDH (IU/L), median (IQR)^ | ^399 (272–587)^ |
| ^Diagnostic test^ | |  |
|  | ^Initial seronegative result^ | ^50 (27.3)^ |
|  | ^Positive PCR^ | ^7/29 (24.1)^ |
| ^Clinical manifestations^ | |  |
|  | ^Hepatitis^ | ^118 (64.5)^ |
|  | ^Isolated febrile illness^ | ^44 (24.0)^ |
|  | ^Pneumonia with/without hepatitis^ | ^17 (9.3)^ |
|  | ^Meningoencephalitis^ | ^3 (1.6)^ |
|  | ^Pericarditis^ | ^1 (0.5)^ |
| ^Case classification*^ | |  |
|  | ^Confirmed^ | ^116 (63.4)^ |
|  | ^Probable^ | ^67 (36.6)^ |
| ^Follow-up after primary infection^ | |  |
|  | ^Serological test ≥3^ | ^75 (41.0)^ |
|  | ^Serological test ≥4^ | ^43 (23.5)^ |
|  | ^Echocardiography^ | ^113 (61.7)^ |
|  | ^6-month follow-up^ | ^44 (24.0)^ |
| ^Prognosis^ |  |  |
|  | ^Chronic Q fever after primary infection^ | ^0 (0)^ |
|  | ^Treatment duration (day), median (IQR)^ | ^14.0 (9.8–17.0)^ |

^*^ Confirmed or probable cases were classified using the laboratory criteria with clinical evidence of *C. burnetii* infection

**Comment #3**

In the same way, the authors say that 67 patients achieved the diagnosis of probable Q fever. Did clinicians establish a clinical diagnosis and proper treatment in these cases? Did this subgroup of patients undergo to a correct clinical and serological follow up? In this sense it could be useful to use the criteria and recommendations wich appear in the reference 22 by Eldin et al.

**Authors’ response to comment #3>**

As mentioned above, we have clearly defined probable or confirmed acute Q fever. Cases with clinical evidence of *C. burnetii* infection and a laboratory probable result were classified as having probable acute Q fever. The laboratory criteria for probable acute Q fever were a single IgG titer to phase II antigen ≥1:256. Therefore, probable Q fever cases did not undergo serological follow-up. In this study, appropriate antibiotic treatment, including doxycycline, trimethoprim-sulfamethoxazole, fluoroquinolone, or macrolide antibiotics, was performed in 158 (86.3%) cases of acute Q fever and 53 (79.1%) cases of probable acute Q fever.

We have added the following paragraph in the *Clinical characteristics of patients with acute Q fever* subsection in the Results section:

“Appropriate antibiotic treatment, including doxycycline, trimethoprim-sulfamethoxazole, fluoroquinolone, or macrolide antibiotics, was administered in 158 (86.3%) cases, and the median duration of antibiotic treatment was 14.0 days (IQR 9.8–17.0 days). None of the patients with acute Q fever progressed to persistent localized infection or died.”

**Comment #4**

Regarding clinical form of presentation it could be of a high interest to know if the presentation as hepatitis, isolated fever or pneumonia was more frequent depending on the living area of patients, as it happens in other countries like Spain, like it is shown in the reference 20 by Alende-Castro et al.

**Authors’ response to comment #4>**

Thank you for your detailed comment. We also think that it would be of interest to know this. However, clinical manifestations and presentations did not differ according to the living area of patients in South Korea.

**Comment#5**

Can the authors add some information about the treatment and clinical evolution of patients diagnosed with acute Q fever?

**Authors’ response to comment #5>**

Thank you for your detailed query. We have added some information on the treatment and clinical course of patients with acute Q fever in the *Clinical characteristics of patients with acute Q fever* subsection in the Results section as follows:

“Appropriate antibiotic treatment, including doxycycline, trimethoprim-sulfamethoxazole, fluoroquinolone, or macrolide antibiotics, was administered in 158 (86.3%) cases, and the median duration of antibiotic treatment was 14.0 days (IQR 9.8–17.0 days). None of the patients with acute Q fever progressed to persistent localized infection or died.”

**Comment #6**

Chronic Q fever cases: since this disease is infrequent it would be of interest if authors could show more information about the reported cases, for example, which type of vascular involvement showed the other cases apart from those 3 with aortic prosthesis related infection?

**Authors’ response to comment #6>**

Thank you for your comment. We obtained additional information that the other six cases pertained to patients with infected aortic aneurysms. Thus, we have added the following sentence in the *Clinical characteristics of chronic Q fever* subsection in the Results section:

“Vascular infection (8 cases, 47.1%) was the most frequent manifestation of chronic Q fever (Table 3). Of these 8 cases, 3 cases had aortic prosthesis-related infections and the other 5 cases had an infected aortic aneurysm. Six (37.5%) patients had endocarditis.”

**Comment #7**

The authors say that they were 9 cases of vascular infection, but Table 3 shows 8.

**Authors’ response to comment #7>**

Thank you for your comment. We have corrected this error in the Results as follows:

“Vascular infection (8 cases, 47.1%) was the most frequent manifestation of chronic Q fever (Table 3).”

**Comment #8**

Did any of the chronic Q fever cases had previous diagnosis of acute Q fever? If so, was it correctly treated?

**Authors’ response to comment #8>**

Thank you for your detailed query. We also think that it would be useful information to readers. However, among the clinical information available, there were no record of diagnosed or treated acute Q fever in the past.

**Comment #9**

Survey: the paragraph regarding survey responses is redundant since most of the information is available in tables, it could be summarized.

**Authors’ response to comment #9>**

Thank you for your detailed comment. We have summarized the paragraph explaining the survey as follows:

“We surveyed 1,226 physicians online, including 243 infectious disease specialists, and the response rate was 8.4%. The questionnaire results are presented in Table 4. Most physicians (80.6%) worked in university-affiliated hospitals, and 86.4% of respondents had experience performing Q fever tests. However, 38.7% of respondents had no experience in diagnosing Q fever, and 35.0% of respondents had only experience of one or two cases of Q fever. When patients were suspected of having acute Q fever, 55.3% of respondents rarely conducted a second serological test in the convalescent stage if the sera from the acute stage was negative. With regard follow-up clinical assessments and serological tests repeated over 6 months to identify cases of chronic Q fever after acute Q fever, 40.8% of respondents stated that clinical assessments and serological tests were not repeated after primary infection, while 22.3% of respondents repeated clinical assessments and serological tests. The other questions and their results are presented in S2 Table.”

**Reviewer #3**

**Comment #1**

The results are described very clearly in the text and in the tables and figures.

**Authors; response to comment #1>**

Thank you for your thoughtful comment.

**Conclusions**

**Reviewer #1**

**Comment #1**

This work reports the description of cases of Q fever in Korea which is a country for which this disease is not well known, some elements are contributory for the scientific community and others less obviously interesting. From this point of view, surveillance to assess the interest and knowledge of clinicians for Q fever can be removed and is of little interest.

**Authors’ response to comment #1>**

Thank you for your detailed comment. We have removed and summarized the information regarding physicians’ awareness regarding the diagnosis and management of Q fever in the Methods and Results sections.

**Reviewer #2**

**Comment #1**

The authors should make more emphasis in the relevance of their findings, in this sense, the clinical suspicion of Q fever in South Korea could be establish also in the absence of an epidemiological risk factor? Do the authors have any hypothesis to explain that most patients presented as hepatitis? Do the authors consider reevaluating the effect of their intervention (through the survey) in the clinical diary practice regarding Q fever?

**Authors’ response to comment #1>**

Thank you for your detailed query.

1. We have added an important point to emphasize the relevance of our findings in the last paragraph of the Discussion section, as follows:

“Infection can occur in susceptible persons living far from infected animals or contaminated environments because aerosol particles can travel with wind for miles. Therefore, clinicians should suspect Q fever in patients with acute hepatitis of unknown etiology in South Korea. In addition, given the upward trend of acute Q fever cases…”

2. We have added an explanation regarding why hepatitis is the most common clinical manifestation in the third paragraph in the Discussion section, as follows:

“Although there is no obvious hypothesis to explain why most patients with acute Q fever presented with hepatitis in South Korea, a possible explanation can be the significant correlation observed in this study between the number of goat farms and the incidence in human Q fever in South Korea. It is known that hepatitis is more frequent clinical manifestation in sheep- and goat-breeding areas [22]. Although animal sources were not identified in most Q fever cases, the most common kind of animal contact was contact with goats in this study, and there was an increase in the number of goat farms during the study period.”

3. We have shared the survey results with the KCDC. We are collaborating with the KCDC in preparing Korean guidelines to promote the diagnosis and management of Q fever using the findings of this study.

**Comment #2**

The authors refer again to the fact that 27.3% of acute cases tested negative, PCR test were performed only in 15.8% and 36.6% did not undergo a second test. As it was recommended above, this paragraph should be reviewed and clarify all the subgroups of patients considering the diagnosis process.

**Authors’ response to comment #2**

We apologize for the lack of clarity. We have revised the paragraph in the Results and Discussion section. PCR was used in some cases with suspected acute Q fever. Among cases of acute Q fever, PCR (+) cases were seronegative cases in the acute stage. All PCR (+) cases that were seronegative in the acute stage were seropositive in the convalescent stage. Thus, we would like to show that the PCR test might be useful in cases of acute Q fever with initially seronegative results.

Thus, we have revised some paragraphs in the results and discussion sections as follows:

The *Clinical characteristics of acute Q fever* subsection in the Result section:

“Among the patients with acute Q fever, 116 (63.4%) patients underwent serological tests using paired sera collected at least twice during the acute and convalescent phase. Therefore, the remaining 67 (36.6%) cases in which only a single serological test was performed were classified as probable acute Q fever cases. Fifty (27.3%) patients with acute Q fever had seroconversion, from seronegative in the acute stage to seropositive in the convalescent stage. After primary infection, serological follow-ups and echocardiography were performed in 75 (41.0%) and 113 (61.7%) cases, respectively. Positive PCR results for *C. burnetii* were identified in seven cases that showed negative serological results in the acute stage.”

In the discussion section,

“Among the patients with acute Q fever, seroconversion was identified in 27.3% patients through collection of sera at least twice during the acute and convalescent stage; 36.6% patients did not undergo a second serological test during the convalescent stage. Because seroconversion usually occurs 7–15 days after onset of symptoms in acute Q fever, it is necessary to follow-up patients with an antibody test using convalescent serum samples taken 3–6 weeks apart [23]. However, as shown in the results of our survey, more than half of the physicians did not follow-up patients using a serological test. PCR tests can be helpful in the diagnosis of acute Q fever in patients who developed symptoms within 2 weeks; the sensitivity of PCR tests has been reported to be 24%–98% [23]. This study also showed that PCR tests were useful for diagnosing acute Q fever in patients who had negative serological results at the acute stage. Although PCR testing for *C. burnetii* is not widely used to diagnose Q fever in South Korea, its use will enable clinicians to make a correct and early diagnosis of acute Q fever.”

**Comment #3**

What is the availability of Q fever PCR tests in South Korea? In many countries this test is not routinely available and could explain the low percentage of patients in which it was performed. It would also be of interest to describe the indications for PCR test if exist.

**Authors’ response to comment #3**

Thank you for your detailed comment. We have described the availability of Q fever PCR tests in South Korea in the Methods section. The molecular diagnosis was performed using real-time PCR in some individual laboratories. There were no indications for PCR testing, and it was not widely used to diagnose Q fever. As mentioned in our manuscript, the PCR test was useful to diagnose acute Q fever in patients who had negative serological results at the acute stage. Thus, it will enable clinicians to make a proper and early diagnosis of acute Q fever.

**Comment #4**

Regarding chronic Q fever cases, the authors discuss about the risk factors of patients (vascular prosthesis) but they did not explain in the results section if the diagnosed patients had any of these risk factors, both parts of the manuscript should be congruent.

**Authors' response to comment #4**

Thank you for your detailed comment. We have described the risk factors for chronic Q fever in the Results section, as follows:

“However, patients with chronic Q fever were more likely to have congestive heart failure (*P* = 0.001), heart valve insufficiency (*P* < 0.001), prosthetic heart valves (*P* = 0.037), and arterial aneurysms (*P* < 0.001) than patients with acute Q fever.”

**Comment #5**

Finally, the authors could discuss deeply the follow-up of patients in their series and the low relevance that physicians give to this process in light of the answers reflected in the survey.

**Authors’ response to comment #5**

Thank you for your comment. As per your suggestion, we have added the paragraphs in the Discussion section as follows:

“The results of the survey on physician’s awareness indicated that many physicians, including infectious disease specialists, did not know how to correctly diagnose and manage Q fever. This may be due to the nonspecific clinical features of Q fever and the limited diagnostic tools available. Q fever can lead to persistent localized infection in certain patients with risk factors, therefore, it is necessary for clinicians to increase their awareness regarding Q fever to enable correct diagnosis and management.”

**Reviewer #3**

**Comment #1**

The discussion and conclusions are derived from the results of the paper, are comprehensive and clear, and well written.

**Authors’ response to comment #1>**

Thank you for your thoughtful comment.

**Editorial and Data Presentation Modification**

**Reviewer #1**

No response

**Reviewer #2**

In my opinion, the study is valuable but should be reviewed to facilitate its comprehension to readers.

**Reviewer #3**

**Comment #1**

The paper is well written, and the data is presented clearly.

The first paragraph of the introduction needs a reference for the statement that "...and has resulted in large-scaled outbreaks".

**Authors’ response to comment #1>**

We have provided a reference for the statement, as below:

“Schneeberger PM, Wintenberger C, van der Hoek W, Stahl JP. Q fever in the Netherlands - 2007-2010: what we learned from the largest outbreak ever. Med Mal Infect. 2014; 44: 339-353. https://doi.org/10.1016/j.medmal.2014.02.006 PMID: 25108615”

**Comment #2**

The second paragraph in the introduction section, line 5, should be rephrased to "Although the acute Q fever is USUALLY benign with a low mortality rate...", since acute Q fever is not always benign and these cases are well described in the literature.

**Authors’ response to comment #2>**

Thank you for your kind comment. We have added the word “usually” in the sentence.

**Summary and General Comments**

**Reviewer #1**

No response

**Reviewer #2**

**Comment #1**

The authors present an interesting work showing data about the increasing of Q fever cases in South Korea. The overall design of the study is correct, and the results could be of general interest, but some aspects must be clarified.

**Authors’ response to comment #1>**

Thank you for your thoughtful comment.

**Comment #2**

The authors could review some English expression along all the manuscript to improve reading comprehension.

**Authors’ response to comment #2>**

Thank you for your thoughtful comment. We had availed the services of a professional English proofreading service for our manuscript before the previous submission, but unfortunately, it appears that the proofreading was not adequate. We have had the manuscript proofread by a native English speaker again to ensure that the manuscript is free of grammatical and language-related errors.

**Comment #3**

The introduction section could be enriched if some references are added to support data, for example in the first paragraph when the authors refer to large outbreaks.

**Authors’ response to comment #3>**

We have provided a reference for the statement, as follows:

“Schneeberger PM, Wintenberger C, van der Hoek W, Stahl JP. Q fever in the Netherlands - 2007–2010: what we learned from the largest outbreak ever. Med Mal Infect. 2014; 44: 339-353. https://doi.org/10.1016/j.medmal.2014.02.006 PMID: 25108615”

**Reviewer #3**

**Comment #1**

I think the paper is well written and designed.

The importance of the data is mostly local, for the South Korean community, but the paper has also global importance since the issues that are dealt in the paper, about awareness and need for follow up in high risk patients are extremely important everywhere, thus the paper should be accepted and published with the minor revisions I already mentioned.

**Authors’ response to comment #1>**

Thank you for your thoughtful comment.
