# Supplementary material for: Epidemiological investigation and physician awareness regarding the diagnosis and management of Q fever in South Korea, 2011 to 2017
Source: PLoS Negl Trop Dis. 2021 Jun 2;15(6):e0009467. doi: 10.1371/journal.pntd.0009467 (PMC8202952; doi:10.1371/journal.pntd.0009467)
Supplement: Attachment — Submitted filename: Response to comment_20210507.docx [file pntd.0009467.s006.docx]

Manuscript ID: PNTD-D-20-01239

**Title:** Epidemiological investigation and physician awareness regarding the diagnosis and management of Q fever in South Korea, 2011 to 2017

We now resubmit our manuscript titled “Epidemiological investigation and physician awareness regarding the diagnosis and management of Q fever in South Korea, 2011 to 2017” for consideration for publication in the PLOS Neglected Tropical Diseases. We appreciate your acknowledgement of the importance of our manuscript’s subject. We hope that the revised manuscript is now suitable for publication in your journal. We look forward to your response.

Sincerely,

Jung Yeon Heo, MD

Department of Infectious Diseases, Ajou University School of Medicine, 164, Worldcup-ro, Youngtong-gu, Suwon 16499, Republic of Korea.

Tel: 82-31-219-7818; E-mail address: jyeon78@naver.com

**Comment #1**

Please include the title page at the beginning of your manuscript file that lists full author names and institute addresses. This should not be uploaded as a separate file.

**Authors’ response to comment #1>**

Thank you for your comment. We have included the title page at the beginning of manuscript file that lists full author names and institute addresses.

**Comment #2**

Please include the initials, alongside each funding source, of each author to receive each grant.

**Authors’ response to comment #1>**

Thank you for your comment. We have included the initial of author who received the grant, as follows:

“This work was supported by a grant from the Korean Centers for Disease Prevention and Control (No. 2018E230100 to JYH). The funder had no role in study design, data analysis, or preparation of the manuscript.”
